# Injury Epidemiology in Brazilian Rugby Union: Implications for Strength and Conditioning Practice

**DOI:** 10.3390/sports13080247

**Published:** 2025-07-26

**Authors:** Joana Magalhães Marrese, Tamiris Beppler Martins, Mark Russell, Rodrigo Okubo

**Affiliations:** 1 Department of Physiotherapy, Santa Catarina State University, Florianopolis 88080-350, SC, Brazil; joanamagmar@gmail.com; 2 Department of Physiotherapy and Physical Therapy Graduate Program, Santa Catarina State University, Florianopolis 88080-350, SC, Brazil; tamiris.martins@udesc.br; 3 School of Sport and Wellbeing, Faculty of Health, Wellness and Life Sciences, Leeds Trinity University, Leeds LS18 5HD, UK; m.russell@leedstrinity.ac.uk

**Keywords:** rugby union, sports injuries, injury prevention, strength and conditioning, athletic performance, epidemiology

## Abstract

Rugby Union is a high-impact sport with considerable injury risk, especially in emerging rugby settings where structured physical preparation may be limited. This study aimed to assess the epidemiological profile and injury incidence among Rugby Union players in Brazil, providing insights to inform strength and conditioning strategies. A cross-sectional observational study was conducted between October 2023 and February 2024 using a digital questionnaire that captured demographic data, sports participation history, and detailed information about injuries sustained in 2022 and 2023. A total of 236 players participated (58.9% male; mean age = 29.4 ± 7.5 years), with males averaging 29.6 ± 7.7 years and females 29.1 ± 7.5 years. Overall, 183 injuries were reported. Most injuries occurred during matches (73.3%) and were contact-related (82.1%), with the shoulder/clavicle and knee being the most affected regions. Ligament injuries (27.3%), dislocations (15.3%), and fractures (16.4%) were the most prevalent types. Female players had a distinct injury pattern, with a greater proportion of non-contact mechanisms. Significant associations were found between injury occurrence and sex (*p* = 0.012), playing modality (*p* < 0.001), injury type (*p* = 0.013), and recovery time (*p* = 0.006). These findings highlight the urgent need for tailored strength and conditioning interventions focused on injury prevention and athletic preparedness. Such programs should address sport-specific demands, promote neuromuscular resilience, and be accessible across competitive levels to improve performance and minimize injury-related setbacks.

## 1. Introduction

Rugby Union is characterized by its intense physicality, involving frequent collisions, tackles, rucks, mauls, and scrums, which significantly increase the risk of injury [1,2]. Scrums alone account for 35% to 51% of catastrophic cervical spine injuries, underscoring the unique risks of Rugby Union compared to Rugby League [1]. Tackles are also among the most injury-prone elements of the game, responsible for 29% to 32% of all reported injuries [1,2], with high-speed tackles—particularly from behind—playing a key role in injury occurrence [3]. Additionally, soft tissue trauma is highly prevalent, with studies reporting that 40% of Rugby Union players in Scotland have required sutures due to injuries sustained during play [4].

According to World Rugby, the sport continues to grow globally, with over 8 million registered players in 2021 and more than 11,000 active participants in Brazil [5]. In Brazil, the most practiced formats are Rugby XV and Rugby Sevens [6], both of which demand high levels of strength, power, speed, endurance, and agility from athletes [7]. These physiological demands highlight the crucial role of structured strength and conditioning (S&C) programs not only in optimizing performance but also in reducing injury risk. Despite this, injury incidence remains high, with match-related injuries occurring at a rate of approximately 81 per 1000 player-hours—comparable to other contact sports [8,9]. Discrepancies across studies in terms of injury definitions, populations, and surveillance methods limit the comparability of data and hinder the development of evidence-based preventive approaches [10,11,12].

Concussions remain a significant concern, as Rugby Union consistently ranks among the sports with the highest rates of head trauma [13]. Around 50% of these concussions occur during tackles, most often affecting the player initiating contact [2,14]. These findings emphasize the urgent need for integrated prevention strategies that combine technical training, appropriate physical conditioning, and, where applicable, rule adaptations [15]. Moreover, the economic burden of injuries—including medical expenses, lost wages for professionals, and reduced player availability—may compromise team cohesion, tactical execution, and overall performance, especially in key competitions [6]. Although correlations between player availability and team success have been established in other elite sports [16,17], their specific effects in Rugby Union are yet to be fully clarified.

Despite its risks, Rugby Union provides moderate-to-vigorous physical activity and is associated with physical, mental, and social benefits [18]. However, injuries can result in long-term absences, undermining individual development and team outcomes. In a growing rugby community such as Brazil, understanding the local injury landscape is vital for informing preventive practices that support both athlete health and high-level performance.

Although previous research has documented general injury trends in Rugby Union, few studies have focused on Brazilian athletes or contextualized their findings within S&C frameworks. By identifying the most prevalent injuries, their mechanisms, and associated outcomes, professionals can design targeted S&C programs that mitigate injury risk and enhance resilience [19,20]. Therefore, this study aimed to describe the epidemiological profile and injury incidence among Rugby Union players in Brazil and to discuss the implications for physical preparation and injury prevention strategies grounded in S&C principles.

## 2. Materials and Methods

### 2.1. Study Design

This study followed an epidemiological, cross-sectional, observational, and descriptive design with a quantitative approach.

### 2.2. Participants

Participants were recruited from physical training facilities, rugby tournaments, athlete social groups, and social media platforms. Inclusion criteria encompassed Rugby Union players (both male and female) active in Brazil during 2022 and 2023, with no restrictions on age or years of experience. Individuals who did not agree to the terms of the Free and Informed Consent Form or who declined to participate were excluded.

### 2.3. Data Collection Procedures

This research was approved by the Ethics Committee for Research Involving Human Subjects at the State University of Santa Catarina (UDESC) (approval number: 6.832.710; CAAE: 78712924.0.0000.0118). Data were collected between October 2023 and February 2024 using a structured digital questionnaire hosted on Google Forms^®^.

The questionnaire consisted of seven sections. In the first, participants reviewed and responded to the informed consent form. Those who declined consent were automatically excluded from the study. The second section collected demographic and sports-related data using a custom registration form developed by the researchers. This included age, sex, body mass, height, playing position in both Rugby XV and Sevens, competition level, and club affiliation. Additionally, items addressed access to healthcare professionals, physical preparation routines, and athletes’ perceptions regarding injury prevention, allowing for indirect insights into strength and conditioning practices.

From the third section onward, questions adapted from the Injury Report Form for Rugby Union [12]—translated and cross-culturally validated for Brazilian Portuguese [21]—were used to identify and describe injuries sustained during training or match play in 2022 and 2023. Only injuries that resulted in absence from at least one full training session or match were considered.

To reduce respondent burden and improve data reliability, the questionnaire limited injury reporting to a maximum of three incidents per participant. Information collected included injury context and severity, rugby modality (XV or Sevens), date of injury, time lost, playing position, injured body region, injury type (e.g., sprain, fracture, concussion), and the setting in which the injury occurred (training, match, or other). Additional variables assessed included injury mechanism (contact vs. non-contact), presence of dangerous play or rule infraction, recurrence, and whether the injury received a professional diagnosis. Injury side (left or right) was additionally recorded, when applicable, to explore potential asymmetry patterns.

Additionally, the questionnaire included open-ended questions aimed at capturing players’ perceptions regarding injury prevention strategies. Responses to these questions were analyzed qualitatively to identify common themes related to physical preparation, recovery practices, and the role of professional support.

### 2.4. Data Analysis

Quantitative data (Supplementary File S1) were exported to Microsoft Excel^®^ Office16 and analyzed using the Statistical Package for the Social Sciences (SPSS^®^) version 20 (IBM Corp., Armonk, NY, USA). Descriptive statistics were presented as means and standard deviations for continuous variables and as absolute and relative frequencies for categorical variables. The Kolmogorov–Smirnov test was used to assess normality. Between-group comparisons of continuous variables were conducted using Student’s *t*-test or Mann–Whitney U test, as appropriate. Associations between categorical variables were assessed using the chi-square test (χ^2^) or Fisher’s exact test for 2 × 2 tables. For comparisons involving more than two categories (e.g., Rugby XV, Rugby Sevens, and both), chi-square tests for independence with post hoc analyses were applied. A 95% confidence interval was adopted for all analyses, with statistical significance set at *p* < 0.05.

## 3. Results

Of the 237 individuals who accessed the questionnaire, one did not provide informed consent and was therefore excluded, resulting in a final sample of 236 participants. The mean age of all participants was 29.4 ± 7.6 years; male participants (58.9%) averaged 29.6 ± 7.7 years, and female participants 29.1 ± 7.5 years. Detailed sample characteristics—including anthropometric data, professional support, competitive level, and playing positions—are presented in Table 1.

### 3.1. Injury Incidence and Characteristics

A total of 183 injuries were reported between 2022 and 2023. These occurred predominantly during matches (73.3%), with contact being the leading mechanism (82.1%). Injuries in Rugby XV were more frequent than in Sevens (62.3% vs. 37.7%). Lateralization showed a slight predominance of left-side injuries (48.6%). Recovery time varied, with most athletes returning within one to three months (30.6%), though 10.4% had not returned at the time of data collection (Table 2).

Interestingly, when analyzing the broader sample (n = 236), players who exclusively practiced either XV or Sevens had a significantly higher injury rate (46.2% and 46.4%, respectively) compared to those who played both codes (15.2%). Although these data must be interpreted cautiously, they suggest that exposure to both formats might be associated with lower injury rates, potentially due to training adaptation or selection factors. 

Recurrent injuries were reported by 8.1% of participants, and 14.8% required more than four months of recovery. Ligament injuries, dislocations, and fractures were among the most severe, while muscle injuries were associated with shorter recovery times.

### 3.2. Injury Distribution and Mechanisms

Wings had the highest injury rate (64%, n = 32), followed by hookers (29%, n = 9) and fly-halves (24%, n = 7). In contrast, props (14%, n = 6) and scrum-halves (13, n = 4) had the lowest rates, suggesting differences in exposure to contact and game dynamics by position.

A total of 183 injuries were reported by 124 athletes, indicating that some individuals experienced more than one injury during the study period. In total, 150 (82.0%) were contact-related and 33 (18.0%) non-contact. Among males, contact injuries accounted for 85.1%, while females had a relatively lower proportion (76.6%), indicating a distinct injury mechanism pattern by sex. The most frequent mechanisms were being tackled (45.5%, n = 79) and tackling (23.4%, n = 40), which together accounted for nearly 70% of injuries. Rucks (13.0%, n = 23) and general collisions (10.4%, n = 18) were also notable contributors. Less common causes included scrums, acceleration, directional changes, and stepping on irregular terrain—often related to a lack of proprioceptive control or insufficient preparatory training.

The most commonly injured regions were the knee (32.1%, n = 58), shoulder/clavicle (18.4%, n = 33), and ankle (14.7%, n = 26). Injuries involved ligament ruptures (27.3%, n = 50), dislocations (15.3%, n = 28), and muscle tears (16.4%, n = 30), especially in high-impact and load-bearing joints. 

To ensure clarity and avoid overinterpretation of low-frequency findings, we focused on reporting injury mechanisms and anatomical regions with a relative frequency of at least 10%, as patterns below this threshold may be more susceptible to random variation in this sample.

### 3.3. Associations with Injury Occurrence

Statistical analysis revealed significant associations between injury occurrence and sex (*p* = 0.012), as well as playing modality (*p* < 0.001). Males were more frequently injured, and Rugby XV was associated with higher injury rates. A further significant association (*p* = 0.001) indicated that female athletes were more represented in Sevens, which may partially explain differing injury patterns. These findings are summarized in Table 3.

Recovery time was significantly associated with injury type (*p* = 0.001). Ligament injuries had the longest recovery periods, while muscle injuries were more likely to result in brief absences. These results reinforce the importance of individualized strength training and targeted prevention protocols based on injury type.

Additionally, a chi-square analysis revealed that female athletes were more represented in Sevens (49.0%) compared to XV (33.1%), whereas male athletes were more prevalent in XV (66.9%) than in Sevens (51.0%) (χ^2^ = 12.331). This sex-based distribution across modalities may help explain some of the observed differences in injury incidence.

### 3.4. Injury Characteristics by Rugby Code

When stratifying injuries by the rugby code in which they occurred, no statistically significant differences were found regarding injury type (*p* = 0.6079), body region affected (*p* = 0.1351), or injury mechanism (*p* = 0.4993), as shown in Table 4. This suggests that the injury patterns were similar between Rugby XV and Sevens in this sample. 

### 3.5. Athlete Perceptions on Injury Prevention

Athlete perceptions were obtained through an open-ended question in the questionnaire, which invited participants to describe strategies they considered important for injury prevention in rugby. Among the 236 respondents, 174 athletes (73.7%) provided qualitative responses, which were categorized thematically.

Athletes emphasized that effective injury prevention involves a combination of proper physical conditioning, technical skill development, and professional support. Frequently mentioned strategies included structured strength and conditioning programs, mobility training, rugby-specific simulations, and progressive load management. The presence of S&C coaches was described as essential to achieving adequate readiness for competition.

Other commonly reported practices included recovery protocols (e.g., cryotherapy and rest), dynamic warm-ups, proprioceptive exercises, myofascial release, and weekly flexibility routines. Many athletes also highlighted the importance of learning correct gameplay techniques and adhering to scientifically validated warm-up programs, such as the World Rugby Activate program.

## 4. Discussion

This study aimed to analyze the epidemiological profile and injury incidence among Brazilian Rugby Union players, with particular attention to injury characteristics and associated factors. The results revealed a higher incidence among male players (58.9%), particularly in the Rugby XV format (62.3%). Most injuries occurred during matches (73.3%) and were contact-related (82.1%). Recovery times varied, with one to three months being the most common (30.6%). Positions such as wing, hooker, and center were more frequently associated with injury, consistent with their roles in high-speed actions and repeated physical contacts. As previously described, injuries predominantly affected load-bearing joints like the knee and shoulder/clavicle—regions consistently implicated in rugby-related trauma. Ligament ruptures, dislocations, and fractures were the most common injury types.

In addition to these general patterns, we further examined whether injury characteristics differed between the two rugby codes. Although Rugby XV and Sevens differ considerably in structure, match duration, number of players, and physical demands [22,23], our data—based on the 183 injuries reported in this study—did not reveal statistically significant differences in injury types, affected body regions, or mechanisms between the two codes (Table 4). These findings differ from those reported in previous studies. For instance, Fuller et al. (2016) [22] found that injury burden in Rugby Sevens was two to three times higher than in Rugby XV for both backs and forwards, potentially due to the higher ball-in-play time, reduced team size, and increased match intensity [24].

Moreover, Rugby Sevens players are exposed to greater physiological stress and fatigue due to shorter recovery intervals, broader positional responsibilities, and a faster-paced style of play [23], all of which—combined with frequent tackling actions—are known to contribute to higher injury susceptibility [25,26]. Despite these documented differences, our sample may have lacked sufficient power or heterogeneity to detect such disparities—especially given the inclusion of a broad mix of competitive levels.

Interestingly, exploratory analysis of our full dataset (n = 236) indicated a higher injury rate among players who exclusively played either XV (46.2%) or Sevens (46.4%) compared to those who played both codes (15.2%). While this finding should be interpreted with caution, it raises hypotheses regarding the potential protective effects of diversified training stimuli, more comprehensive physical preparation, or self-selection biases among dual-code players. These insights warrant further investigation and may inform the design of more tailored S&C programs. 

These findings are consistent with previous studies indicating that Rugby Union poses a high risk of injury due to frequent collisions and intense physical engagement, especially during matches [27]. The biomechanics of common rugby actions—tackling, rucking, and scrummaging—place considerable load on joints and soft tissues, predisposing players to acute injuries [28,29]. Higher injury rates observed in positions such as wings, hookers, and fly-halves are aligned with their functional roles: high-speed running, repetitive contact, and tactical demands. Prior studies also report differential injury patterns between forwards and backs, reinforcing the need for position-specific S&C strategies [1,3].

The elevated incidence among hookers and wings may be linked to their frequent exposure to high-speed collisions and tactical responsibilities involving open play and finishing actions. Although tackling was the leading cause of injury overall, hookers are specifically exposed to scrums and rucks, which—despite accounting for a smaller proportion of injuries in this study (13% and lower, respectively)—still contribute to cumulative upper body stress in this position. Mauls, another high-impact phase often involving hookers, were not categorized separately in our questionnaire but are likely contributors to shoulder and cervical overload. These data highlight the importance of targeted conditioning programs aimed at increasing joint stability, upper body strength, and injury resilience, particularly in collision-prone roles [30].

Although concussions are among the most commonly reported injuries in Rugby Union globally [6,31,32], they were less frequent in this study. This discrepancy may reflect underreporting, lack of symptom awareness, or cultural factors where players underreport injuries to avoid removal from play [33]. Educational initiatives, sideline protocols, and stricter enforcement of concussion guidelines are essential for athlete safety [34,35], but should be integrated with S&C practices that include neck strengthening, proprioception, and safe contact training.

The predominance of injuries during legal plays, where no rule infraction was identified by referees, underscores the inherently risky nature of rugby—even under standard regulations. Rule changes such as lowering tackle height and adjusting ruck dynamics have shown promise in reducing injury incidence [36], but must be coupled with evidence-based physical preparation. Structured warm-ups, progressive overload, contact conditioning, and recovery optimization are key components of a modern injury prevention model in rugby [27].

Notably, this study found a greater injury incidence in Rugby XV compared to Sevens, particularly among male players. While rules and field size are consistent across sexes, physiological demands and player density differ. These findings suggest that sex- and modality-specific conditioning strategies may be necessary. The literature has also pointed to sex-based physiological differences (e.g., cardiorespiratory capacity and muscle fiber distribution) that influence injury risk and recovery profiles [37,38].

The impact of injury on training continuity and competitive participation was evident, with over 30% of players being absent for up to three months and nearly 15% exceeding six months of recovery. These prolonged absences are especially relevant for injuries such as ligament tears, which often require structured rehabilitation and gradual return to play. Evidence shows that team performance is inversely related to injury burden [30], emphasizing the strategic importance of injury prevention in high-performance planning.

These additional analyses provide a more nuanced understanding of injury patterns in Brazilian rugby athletes, especially in light of differences between XV and Sevens codes. Although no significant differences were found in injury types or mechanisms between the two formats, we identified that players who practiced both codes had significantly lower injury rates, suggesting potential protective effects of diversified physical exposure. Furthermore, the stratification by sex and level of play contributes to a more contextualized interpretation of the data, which is crucial for tailoring injury prevention and S&C strategies. Finally, the qualitative insights gathered reinforce that effective prevention must combine physical preparation, technical skill, and professional guidance. Together, these findings offer actionable knowledge for practitioners supporting rugby athletes.

### 4.1. Study Limitations

Despite the relevant findings, some limitations should be considered. The cross-sectional design does not allow for causal inference, and the use of self-reported data may introduce recall bias. Voluntary participation could lead to selection bias, as athletes with prior injuries may have been more inclined to respond. The number of injuries per athlete was limited to three to avoid recall bias and reduce survey fatigue. As a result, the total injury count may be underestimated for athletes with more frequent occurrences. Additionally, the absence of direct verification of reported injuries limits clinical precision. Future studies should use longitudinal designs, include physical assessments, and stratify findings by competitive level and training background.

### 4.2. Clinical and Practical Implications

The results offer clear guidance for professionals involved in injury prevention and performance enhancement in Rugby Union. Preventive strategies should prioritize position-specific S&C interventions, focusing on strength, power, joint stability, neuromuscular control, and reactive capacity. Warm-up and mobility protocols should be standardized and monitored for adherence. Rehabilitation plans must be individualized and include return-to-play testing, load monitoring, and psychological readiness.

In parallel, strategies to reduce underreporting of concussions must include educational programs and reinforcement of medical protocols. Collaboration among physiotherapists, coaches, medical staff, and S&C coaches is vital to ensure athlete safety and continuity. Optimizing training environments through evidence-based S&C programming can minimize injury risk, enhance performance, and promote career longevity in rugby athletes.

## 5. Conclusions

This study presented a comprehensive overview of the injury profile among 236 Brazilian Rugby Union players, documenting 183 injuries reported between 2022 and 2023. Injuries occurred predominantly in the XV modality and during match play, with contact-related mechanisms being the most frequent. The knee and shoulder/clavicle regions were the most commonly affected, and ligament injuries, fractures, and dislocations were the most prevalent diagnoses. Male players exhibited a higher injury rate, and certain positions—such as hooker and center—were associated with greater exposure to injury.

Qualitative findings emphasized that effective injury prevention depends on a combination of adequate physical preparation, technical skill development, body awareness, and access to professional guidance. These insights reinforce the importance of integrating structured S&C programs into training routines, tailored to positional demands and individual risk profiles.

This study provides updated insights into the injury epidemiology of Brazilian rugby union players across different competitive levels and codes. Our findings underscore the importance of contextualized injury surveillance and support the development of targeted strategies by S&C professionals. The lower injury rate observed among dual-code athletes raises relevant hypotheses for future research and suggests that diversified exposure or training variability may play a role in injury prevention. These results can inform tailored S&C interventions aimed at reducing injury burden and optimizing athletic performance in rugby.

## Figures and Tables

**Table 1:** Sample characterization data.

Variables	Total Sample (n = 236)
**Age** (years) ^X ± SD^ [Mín–Max]	29.4 ± 7.5 [18–53]
**Sex** ^f (%)^	
Male	139 (58.9%)
Female	97 (41.1%)
**Body mass** (kg) ^X ± SD^ [Mín–Max]	80.8 ± 18.9 [50.0–160.0]
**Height** (cm) ^X ± SD^ [Mín–Max]	171.4 ± 8.9 [151.0–194.0]
**Follow-up by health professionals** ^f (%)^	
No follow-up	57 (24.1%)
Biomedical professional	1 (0.42%)
Physiotherapist	109 (46.1%)
Medical doctor	54 (22.8%)
Nutritionist	67 (28.3%)
S&C coach	137 (58%)
Psychologist	54 (22.8%)
**Competition level** ^f (%)^	
Not applicable	12 (5.1%)
Regional/State	55 (23.3%)
National	115 (48.7%)
International	54 (22.9%)
**Position played in XV** ^f (%)^	
Flanker	25 (10.6%)
Fly-half	18 (7.6%)
Fullback	2 (0.8%)
Hooker	18 (7.6%)
Inside Center	19 (8.1%)
Lock	17 (7.2%)
Number 8	3 (1.3%)
Outside Center	14 (5.9%)
Prop	42 (17.8%)
Scrum-half	30 (12.7%)
Wing	19 (8.1%)
I do not play XV	29 (12.3%)
**Position played in Seven** ^f (%)^	
Center	24 (10.2%)
Fly-half	19 (8.1%)
Hooker	41 (17.4%)
Prop	74 (31.4%)
Scrum-half	37 (15.7%)
Wing	14 (5.8%)
I do not play Seven	27 (11.4%)

Legend: X = mean; SD = standard deviation; Min = minimum; Max = maximum; f = absolute and relative frequency.

**Table 2:** Absolute and relative frequency of reported injuries.

Variables	Value
**Reported injury occurrence** ^f (%)^	
Yes	183 (52.5%)
No	112 (47.5%)
**Between modalities** ^f (%)^	(n = 183)
XV	114 (62.3%)
Sevens	69 (37.7%)
**Injury laterality** ^f (%)^	
Not applicable	21 (11.5%)
Right	73 (39.9%)
Left	89 (48.6%)
**Injury occurrence timing** ^f (%)^	
Post-match stretching	1 (0.5%)
Warm-up	3 (1.6%)
Match	134 (73.3%)
Training	45 (24.6%)
**Injury due to contact** ^f (%)^	
No	51 (17.9%)
Yes	132 (82.1%)
**Recurrent injury** ^f (%)^	
Not applicable or unsure	155 (65.6%)
Yes, same injury more than once	19 (8.1%)
No, only occurred once	62 (26.3%)
**Average time away from practice** ^f (%)^	
Does not remember	2 (1.1%)
Less than a week	9 (4.9%)
Between one and four weeks	43 (23.5%)
Between one and three months	56 (30.6%)
Between four and six months	27 (14.8%)
More than six months	27 (14.8%)
Has not returned yet	19 (10.4%)

Legend: f = absolute and relative frequency.

**Table 3:** Comparison of demographic and training characteristics between athletes who reported at least one injury (n = 124) and those who reported no injuries (n = 112).

Variables	Injured (n = 124)	Not Injured (n = 112)	*p*-Value
Age (years) ^X ± SD^	28.5 ± 7.8	29.4 ± 7.7	0.506
Body mass (kg) ^X ± SD^	81.7 ± 17.8	79.9 ± 20.1	0.461
Height (cm) ^X ± SD^	172.7 ± 8.7	170.0 ± 9.0	0.256
BMI (kg/m^2^) ^X ± SD^	27.2 ± 4.6	27.4 ± 5.2	0.327
**Sex** ^f (%)^			**0.012**
Male (n = 139/58.9%)	83 (35.2%)	56 (23.7%)	
Female (n = 97/41.1%)	41 (17.4%)	56 (23.7%)	
**Modality** ^f (%)^			**<0.001**
Sevens	49 (25.7%)	0	
XV	75 (39.4%)	0	
No injury	0	112/53.2%	

Legend: X = mean; SD = standard deviation; BMI = body mass index; f = absolute and relative frequency; *p* = *p*-value; χ^2^ = chi-square.

**Table 4:** Injury characteristics according to the rugby code (XV vs. Sevens).

Injury Variables	*p*-Value
Injury type × Code	0.6079
Body region × Code	0.1351
Injury mechanism × Code	0.4993

Legend: Association between injury characteristics and the rugby code in which the injury occurred (XV or Sevens). Chi-square test.

## Data Availability

The original contributions presented in this study are included in the article/Supplementary Material. Further inquiries can be directed to the corresponding author.

## Supplementary Materials

The following supporting information can be downloaded at https://www.mdpi.com/article/10.3390/sports13080247/s1, Supplementary File S1: Dataset_RugbyUnion_Study.

## Abbreviations

The following abbreviations are used in this manuscript:

S&C	Strength and Conditioning
SPSS	Statistical Package for the Social Sciences
BMI	Body Mass Index
SD	Standard Deviation
